# Unravelling the formation of carbyne nanocrystals from graphene nanoconstrictions through the hydrothermal treatment of agro-industrial waste molasses†

**DOI:** 10.1039/d4na00076e

**Published:** 2024-03-28

**Authors:** Sampathkumar Jeevanandham, Dakshi Kochhar, Omnarayan Agrawal, Siddhartha Pahari, Chirantan Kar, Tamal Goswami, Indra Sulania, Monalisa Mukherjee

**Affiliations:** a Amity Institute of Click Chemistry Research and Studies, Amity University Uttar Pradesh Noida 201301 India mmukherjee@amity.edu; b Department of Chemical Engineering & Applied Chemistry 200 College Street Toronto ON M5S 3E5 Canada; c Amity Institute of Applied Science, Amity University Kolkata Kolkata West Bengal 700135 India; d Department of Chemistry, Raiganj University Uttar Dinajpur Raiganj West Bengal 733134 India; e Inter University Accelerator Centre Vasant Kunj New Delhi Delhi 110067 India

## Abstract

The delicate synthesis of one-dimensional (1D) carbon nanostructures from two-dimensional (2D) graphene moiré layers holds tremendous interest in materials science owing to its unique physiochemical properties exhibited during the formation of hybrid configurations with sp–sp^2^ hybridization. However, the controlled synthesis of such hybrid sp–sp^2^ configurations remains highly challenging. Therefore, we employed a simple hydrothermal technique using agro-industrial waste as the carbon source to synthesize 1D carbyne nanocrystals from the nanoconstricted zones of 2D graphene moiré layers. By employing suite of characterization techniques, we delineated the mechanism of carbyne nanocrystal formation, wherein the origin of carbyne nanochains was deciphered from graphene intermediates due to the presence of a hydrothermally cut nanoconstriction regime engendered over well-oriented graphene moiré patterns. The autogenous hydrothermal pressurization of agro-industrial waste under controlled conditions led to the generation of epoxy-rich graphene intermediates, which concomitantly gave rise to carbyne nanocrystal formation in oriented moiré layers with nanogaps. The unique growth of carbyne nanocrystals over a few layers of holey graphene exhibits excellent paramagnetic properties, the predominant localization of electrons and interfacial polarization effects. Further, we extended the application of the as-synthesized carbyne product (Cp) for real-time electrochemical-based toxic metal (As^3+^) sensing in groundwater samples (from riverbanks), which depicted superior sensitivity (0.22 mA μM^−1^) even at extremely lower concentrations (0.0001 μM), corroborating the impedance spectroscopy analysis.

## Abstract 2

The hydrothermal synthesis of carbyne nanocrystals within nanoconstricted 2D graphene moiré layers using agro-industrial waste molasses as the carbon source.

## Graphical abstract

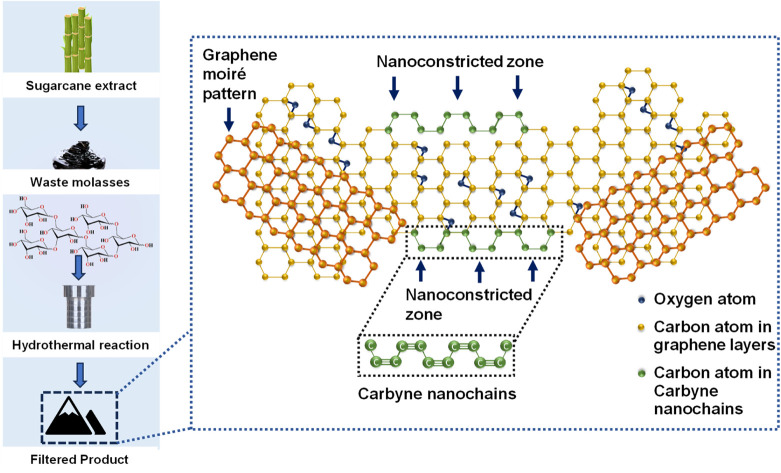

## 1. Introduction

Inspired by monolayer thick graphene,^1^ the controlled synthesis of sp–sp^2^ hybridized monolayer nanostructures acknowledging certain extra degree of freedom fetch paradigm shift towards new age 2D nanomaterials^2,3^ that has attracted great attention among scientists. The ‘flat-and-twist’ nature of graphene moiré layers has several significant characteristic properties, including sizeable band gaps,^4^ giant electron delocalization,^5^ superconductivity,^6,7^ interfacial charge transfer and surface reactivity.^8,9^ More precisely, the presence of vital defects and edge sites acts as building blocks in the design of customizable 2D van der Waals (vdW) heteronanostructures *via* the deterministic assembly of multiple layers possessing weak interlayer interactions.^10,11^ Integrating sp^2^-hybridized 2D graphene moiré layers with 1D sp-hybridized carbyne nanostructures features a scope beyond parent graphene for a plethora of applications owing to their exceptionally manipulable structural modifications.^12,13^ Such an intricate growth of 1D nanostructures constructed in linear atomic arrangement from 2D carbon rich-nanostructures with extended sp^2^-hybridization such as graphene, carbon nanotubes (CNTs), and fullerenes^14^ exhibits excellent physico-chemical properties.^15^

Carbyne, the unique yet much unexplored reactive intermediate of carbon species,^16–18^ lays the foundation for the generation of 1D sp-carbon linear chains, mostly exhibiting closer similarity with graphene and diamonds.^19^ Typically, the design of carbyne nanochains requires precise control over reaction conditions,^19–21^ where its formation is assisted *via* the support of a metal^19,22^ or metal complexes^21^ in an aqueous solution. The rapid functionalization of carbyne chains has also been a key strategy for the generation of stable reactive moieties, releasing distinct free radicals^21^ and constructing other intermediates with atomic reorientation. However, efforts to form a more stable growth of carbyne chains have often relied on completely confined growth within CNTs where precisely controlled growth offers tunability in optical and electronic properties.^23^ Additionally, there is a persistent demand for more simple yet stable synthetic strategies for the generation of carbyne.

Interestingly, several attempts have been made since 2004 to chemically synthesize finite-length carbyne chains in the solution phase using high-temperature experimental conditions like the laser ablation technique (LAL method), especially by utilizing predefined templates such as nanostructured carbon films, polymers or other metal deposited carbon matrices.^24–28^ Pan *et al.*, for the first time, synthesized the finite length carbyne nanochains consisting of alternate single and triple bonds under the LAL method.^29^ Also, Cao *et al.* recently reported the experimental evidence for the formation of kinked carbon chains and interchain van der Waals interactions in carbyne nanocrystals.^30^ Intriguingly, Chalifoux *et al.* described the synthesis of the longest series of conjugated polyynes as a potential model for determining the sp-hybridized carbon atoms in a linear fashion.^25,31^ On the other hand, by using the most familiar graphene layers or CNTs as the sp^2^ hybridized templates, numerous attempts have been made to control the formation of linear sp-hybridized carbyne chains within the nanostructured sp^2^ matrices in the presence or absence of metal supports.^32–35^ More specifically, Casari *et al.* explored the formation of sp chains (polyynes and polycumulenes) embedded within the sp^2^ matrix using a facile cluster beam deposition technique.^15,24^ Similarly, Kano *et al.* utilized a clean monolayer graphene with well-dispersed Pt atoms on its surface, which can trap free carbon adatoms and serve as a critical nucleation site for the carbyne chain formation.^36^ Such intricately grown carbyne chains are terminated by a Pt atom, which eventually results in diverse orientations such as straight, curved, and ringed shapes. Henceforth, the coexistence of sp–sp^2^ hybridization attracted enormous interest in the field of material science, physics and chemistry, wherein the stabilization of sp carbon atoms (carbyne) within sp^2^ matrices still remains highly challenging.

Moreover, there have also been reports of structural transformations of carbon, such as energetically controlled removal of carbon chains in a row-by-row fashion from the graphene layers^33^ by Jin *et al.* and the transition of graphene ribbon to a single carbon chain by Chuvilin *et al.*,^34^ suggesting that the graphene bridges or ribbons have the potential to reconstruct freely with the creation of holes in the same plane at closer proximity to each other. Specifically, these phenomena open numerous possibilities for the formation, migration and nucleation of intermediate chain-type configurations, which can be ultimately constrained down to a few-nm or even one-atom thickness. Intriguingly, the edge atom reconstructions *via* continuous migration of adatoms or internal atomic reorientation have majorly led to the formation of the thinnest carbon bridge, which ultimately converts into two or three parallel chains with energetically favourable conformations.^23,37^

Despite the emergence of numerous top-down and bottom-up strategies for the synthesis of carbyne nanochains,^15,21–25^ metal-free or template-free high-throughput synthesis of carbyne nanostructures with effective utilization of sp–sp^2^ hybridized platform remains highly elusive (Table 1). Nevertheless, heterogeneous hydrothermal chemistry offers industrial scale^51^ high-throughput synthesis of nanostructures with controlled structural manipulation under mild operating conditions. Henceforth, we employed a simple hydrothermal route for the first time to transform agro-industrial wastes into carbyne nanocrystals and holey graphene with moiré patterns by a nanoconstriction phenomenon. Herein, the utilization of agro-industrial waste molasses as the potential carbon source establishes next-generation processing of sustainable and recyclable biomass, which remains one of the biggest concerns related to environmental sustainability in the modern era. The growth of carbyne nanocrystals from graphene nanoconstrictions specifies the coexistence of sp–sp^2^ hybridized carbon nanostructures. Carbyne chain formation involves hydrothermal cutting of graphene intermediates at the nanoscale, where the presence of intrinsic planar epoxy functional groups undergoes reorientation, leading to the generation of nanoconstriction regions exhibiting a change in defects and geometry of graphene. In addition, the dominance of localized electrons and interfacial polarization effects of these sp–sp^2^ hybridized nanostructures are significantly applicable to tailorable sensor devices, particularly in the case of electrochemical-based sensing techniques.

**Table 1:** Comparison table depicting the classification, synthetic approach, and properties of carbyne related nanomaterials

Nanomaterial classification	Method of synthesis	Characteristic properties	References
Hexagonal carbyne crystal	Laser ablation in liquid	Opto-electronic	29
Carbyne quasi-crystals	Laser fragmentation	Dipole polarization, electronic properties	38
Carbyne nanocrystals	Laser ablation in liquid	Electronic	39
Metalated carbyne	Surface chemistry	Electronic	40
Carbyne nanocrystals	Laser ablation in liquid	Optoelectronic sensing	41
Confined carbyne	Direct injection pyrolytic synthesis method	Optoelectronic	42
Formation of amorphous carbyne	PVDF with synchrotron radiation	Electronic, energy storage, spintronics	43
Carbyne from graphene	Sputtering process	Conducting channels	44
Carbyne from graphene constrictions	Electron irradiation	Thermoelectric	45
Carbyne on graphene	*In situ* TEM irradiation	Electronic, mechanical	46
Carbyne enriched carbon film	Chemical dehydrohalogenation	Mechanical energy harvesting	47
Carbon atom wire	Nanosecond pulsed laser deposition	Energy storage, electronic	48
Long chain carbyne	Multilevel pulse-voltage injection	Electronic	49
Carbyne material	Low temperature pyrolysis	Optoelectronic	50
Carbyne nanocrystal from graphene nanoconstrictions	Low-temperature hydrothermal carbonization	Dielectric permittivity, opto-electronic and electrochemical sensitivity	Our work

## 2. Materials & methods

Agro-industrial waste molasses were obtained from a sugarcane mill at Shamli (Uttar Pradesh) as well as from Lucknow, India. Double deionized water was used throughout all the experimental conditions.

### 2.1 Synthesis of sp–sp^2^ hybridized graphene nanosheets

Graphene nanosheets (Cp comprising sp–sp^2^ hybridization) were synthesized under hydrothermal conditions using agro-industrial waste molasses as the source. Around 5 mL of the precursor molasses was continuously stirred for 2 h and then transferred to a 40 mL PTFE-lined stainless-steel autoclave. The hydrothermal conditions were set at 200 °C for 4 h, modified from our earlier report.^52^ Subsequently, the resulting crude product, referred to as ‘molasses cake’ (Mc), was subjected to continuous flushing with de-ionized water for 3 h (volume = 500 mL). Commercial dialysis membranes 100–500 Da and 500–1000 Da (Spectrum Lab CE Spectra Por) were utilized for purifying graphene nanosheets from Mc. Comparatively, 500–1000 Da membranes resulted in a higher percentage yield (>70%). The time for rinsing with the dialysis membranes was around 20–30 min, followed by vacuum drying the resultant at 60 °C overnight. Later, around 10 μL of the as-obtained sample was spin-coated on the silicon wafer (area = 1 cm^2^) and vacuum-dried overnight for STM characterization. Solid-state powdered materials were utilized for dielectric measurements.

### 2.2 Morphological characterization

The X-ray diffraction (XRD) patterns were recorded using a Cu Kα radiation (1.5406 Å) M/S Philips X'Pert Pro instrument in the 2*θ* range of 5–60°. High-resolution transmission electron microscopy (HRTEM) images were captured at an acceleration voltage of 200 kV on a JEOL, JEM-2100F electron microscope from the Inter-University Accelerator Centre (IUAC), New Delhi. The samples for HRTEM characterization were prepared by drop casting the material onto a carbon-coated copper grid followed by drying for more than 8 h at room temperature. Scanning Tunneling Microscopy (STM) images were obtained using a multi-mode probe microscopy system with Nanoscope IIIa controller from IUAC, New Delhi.

### 2.3 Chemical characterization

A Raman spectrometer (Varian 7000 FT-Raman and Varian 600 UMA) connected with a microscope was utilized for the analysis. Raman analyses were conducted using integrated micro-Raman-AFM assembly to differentiate the graphene-rich and carbyne-rich regions. FTIR spectra were recorded at a fixed resolution of 4 cm^−1^ in the wavenumber range 4000–500 cm^−1^ using an ATR-FTIR model Nicolet-5DX FTIR spectroscopy instrument. (The samples for FTIR were prepared on glass slides.) All analyses were carried out in Origin Pro 8.1 software.

X-ray photoelectron spectroscopy (XPS) measurements were performed at the BL-14 beamline of the Indus 2 synchrotron radiation source, Indore. XPS measurements were carried out with a double crystal monochromator (DCM) using a pair of Si (111) crystals. The samples for XPS were coated on silicon wafers, and the stoichiometry was determined from the relative areas of C, N, and O peaks.

Beamline specifications: source bending magnet (1.5 T), length of the beamline – 39 m from the tangent point, energy range – 2–15 keV, acceptance – 0.4 mrad (V) × 3.0 (H), pre-mirror – 1.1 m long, Pt coated horizontal toroid shape mirror monochromator DCM using pair of Si (111) crystals, spot size at sample – 1.0 mm (H) × 2.0 mm (V), Detector Phoibos 225 hemispherical analyser with MCP detector photon flux [pH s^−1^] – 10^10^. XPS analysis and deconvolution of the peaks were carried out using XPS peak fit 4.1 software using Gaussian–Lorentzian line shapes. All asymmetric fitting procedures were followed based on the previously reported standard literature.^53–56^

Liquid NMR data were recorded on Bruker Avance AV-III (400 MHz for 1H) at IIT Delhi in CDCl_3_. Solid state NMR was analysed using Bruker, Avance II (500 MHz for ^1^H) at CSMCRI, Bhavnagar. Solid state ^13^C NMR was recorded on JEOL, model ECX 400 at 400 MHz. HRMS was performed on Shimadzu, QP-2010, at IISC, Bangalore. Electron paramagnetic resonance (EPR) spectrometry was analysed using a Bruker Model EMX MicroX instrument at AIRF, JNU, New Delhi. Elemental analysis was performed using a PerkinElmer 2400 Series CHNS/O Analyser.

### 2.4 Dielectric measurements

The solid powder samples in EPR tubes were flushed before use with gaseous helium/liquid nitrogen before insertion into the cryostat and kept in airtight conditions. The measurements were performed in the transmission/reflection network analyzer, Agilent 4294A precision impedance analyzer.

### 2.5 Electrochemical As^3+^ sensing applications

#### Electrode modification using Cp

Cyclic voltammograms (CV) were recorded in a three-electrode cell system at 50 mV s^−1^ using a screen-printed carbon electrode (SPCE) in which the carbon electrode acts as the working electrode, Ag/AgCl reference electrode and a platinum wire as the counter electrode in a 5 mM K_3_Fe(CN)_6_/5 mM K_4_Fe(CN)_6_ solution as redox probe. The SPCE electrode system was rinsed with water and then modified by coating it with Cp; after that, 50 μL of the Cp (2 mg) suspension was drop cast on the SPCE surface and left overnight for drying. The electrochemical investigation was then carried out on the modified electrode to determine various electrochemically assisted sensing parameters. The analytical performance of the modified electrode was assessed by testing its response using different concentrations of arsenic solution, ranging from 0.0001 μM to 100 μM.

#### Effect of concentration study on Cp/SPCE electrode

The effect of As^3+^ concentration on the Cp-coated SPCE was investigated in the range of 0.0001 μM to 100 μM. CV study was conducted in a 5 mM K_3_Fe(CN)_6_/5 mM K_4_Fe(CN)_6_ solution to determine the minimum detectable concentration using the modified electrode at a scan rate of 50 mV s^−1^. Arsenic solutions of different concentrations ranging from 0.0001 μM to 100 μM were drop cast at SPCE containing the Cp-modified electrode. This process was followed for all different concentrations of As^3+^ and electrodes were washed each time before proceeding with the electrochemical experiments.

#### Electrochemical impedance spectroscopy (EIS) analysis of Cp

The electrochemical performance exposed to arsenic was studied by electrochemical impedance spectroscopy (EIS). The EIS analysis is generally conducted by utilizing an equivalent circuit model that is tailored to the electrochemical properties of Cp deposited over the SPCE. The experiment was performed using varying concentrations of As^3+^ solution (electrolyte) ranging from 0.0001 μm to 100 μM. The *Z*′ and *Z*′′ values in the graph correspond to the real and imaginary parts of the impedance spectrometric analysis, respectively. The semicircle parameters represent the electron transfer resistance (width) and the double layer capacity (height of the semicircle) of the film.

#### Real-time electrochemical sensing using groundwater samples (from riverbanks)

The platinum (Pt) electrode was purchased from Gamry Instruments, USA, with a geometrical surface area of ∼0.071 cm^2^ (3 mm in diameter) and was utilized for real-time electrochemical sensing. Prior to use, the Pt-electrode was polished with 0.1 mm, 0.3 mm, and 0.5 mm of alumina/DI water slurries on a polishing cloth to obtain a clean surface. Then, the polished electrode was rinsed thoroughly with ethanol, DI water and acetone in an ultrasonic bath. Cp was coated on the Pt electrode (Pt–Cp) by taking 1 mg per mL carbyne powder and dispersing it in DI water. The dispersion was electrochemically coated onto a polished Pt-electrode *via* cyclic voltammetry (CV) by scanning within a potential window between −0.1 V to 1 V with a scan rate of 10 mV s^−1^ for 10 cycles. Once the voltammogram was almost stable, the electrode was taken out and kept for drying. All voltammetric measurements were carried out in a three-electrode cell using Gamry Reference 600 Potentiostat/Galvanostat/ZRA, where a Pt-wire was taken as a counter, an Ag/AgCl electrode as a reference and Pt–Cp as a working electrode. Groundwater samples were obtained from the tributary of Yamuna river, (*i.e.* Hindon river traversing through Saharanpur, Muzaffarnagar, Meerut, and Baghpat districts in Uttar Pradesh, India) exceeding the desirable limit of As^3+^ concentration (more than 0.05 mg L^−1^). For every trial, 20 mL of the river water sample was evaluated for As^3+^ toxicity.

### 2.6 Molecular dynamics simulations

To investigate the mechanism involved in the production of carbyne chains formed from the nanoconstricted regions of holey graphene, we have resorted to density functional theoretical (DFT) and molecular dynamics (MD) simulations. The decomposition of holey graphene to carbyne has been modelled by ReaxFF^57^ reactive force field-based MD simulation. For an accurate description of the chemistry of hydrocarbons, ReaxFFCHO has been developed from an extensive analysis of a good number of training sets of atomic charges, bond lengths, bond, valence and torsion angle energies,^58^ heats of formation, and various hydrocarbon reaction energies, and has been utilised to explain a plethora of phenomena such as the oxidation of graphite, combustion and pyrolysis of coal and other hydrocarbons *etc.* A good account of the performance of this force field, compared against DFT, can be found in ref. 59. A good agreement between DFT and MD with the ReaxFFCHO force field encouraged us to utilise ReaxFF MD simulation to study the thermal decomposition of holey graphene for a larger graphene chunk than a smaller model for which DFT could be performed. We conducted a 200 ps NVT simulation at 2000 K temperature, which is much higher than the experimental temperature, to make the simulation accessible within a 200 ps timescale (Fig. 6a). It is also to be noted that during the experiment, the graphitic material did not encounter such high temperatures due to the dissipation of heat.^60^ A Berendsen–Anderson temperature thermostat with a damping constant of 100 fs was used during the simulation and the timestep was kept at 0.2 fs. Post-processing of the MD output is carried out through Fortran programs, and the visualizations are done by Visual Molecular Dynamics (VMD) software.^61^

A finer insight into the mechanism of carbyne chain formation from the zigzag end was gained from density functional theoretical (DFT) calculation on a model system consisting of a single polyacene chain capped by a larger benzenoid system from two ends. For the sake of minimising the complexity of the computation, the end benzene rings were hydrogenated except for the central polyacene system. The system is represented in Fig. S4a.† The DFT calculation was done in the Quantum Espresso suite of software^62^ using the ultrasoft Vanderbilt pseudopotential method with BLYP exchange–correlation function.^63^ The Monkhorst–Pack scheme is used for sampling the Brillouin zone.^64^ On subsequent optimization, the formation of two single carbon chains was realized, and the alternative single and triple bonds in Fig. S4b† represent a carbyne chain formation.

A single-layer rectangular graphene carved from the longer end confined in a 75 Å × 35 Å × 40 Å periodic box has been used to construct our simulation system (Fig. S5†). The cut-in from the longer end has been made to model the nano-confinement generated in such holey graphene sheets. The system was first energy minimized and then used for NVT MD simulation. The MD simulation was carried out using the LAMMPS atomic simulation package.^65^

The various steps of the formation of carbyne chains from holey graphene strips modelled by molecular dynamics simulation are presented in Fig. 6a. It can be readily observed that the number of bonds broken increases with time and thereby facilitates the formation of single carbon chains. This can be envisaged from the plot of the potential energy of the entire system with time (Fig. S3a†). The number of bonds broken is also calculated from the post-processing of the MD output by programs developed by our group. The percentage of broken bonds is presented in Fig. S3b,† which clearly advocates for the observed trends in the reaction path. The mechanism of the process also dictates that the bond breaking takes place preferably from the zigzag ends to make carbyne chains.

It has been a matter of large debate whether the carbyne chains are preferably linear or kinked. Although kinked structure has been predicted for carbyne chains by laser ablation in liquid (LAL) from graphite,^29^ electron irradiation on graphene membranes also reports the preferential formation of bent carbon chains (kinked) under conditions of low density.^34^ DFT calculations have shown that the carbon chains have the least effect of bending on the strain energies, thus advocating their high flexibility to form bent/kinked structures.^66^ Our DFT result also indicates that short carbon chains are preferably formed from the edges in conditions of constriction and are preferably bent in the optimized structure. Molecular dynamics simulation also suggests that the flexibility of the carbyne chains leads to the formation of wavy chains (circled in red in Fig. 6a).

## 3. Results and discussion

### 3.1 Formation of sp–sp^2^ hybridized configuration in holey graphene nanosheets

**Fig. 1:**
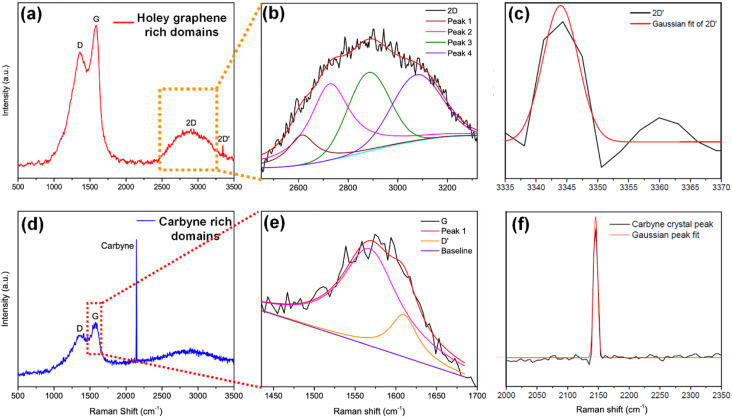
(a) Raman spectrum of Cp with a holey graphene-rich region. (b and c) Deconvoluted spectrum of the 2D and 2D′ band corresponding to (a). (d) Raman spectrum of Cp with a carbyne-rich region. (e) Deconvoluted spectrum of the G/D′ band along with (f) characteristic carbyne peak corresponding to (d).

To elucidate a clear insight into the emergence of 1D carbyne nanocrystals from nano-constricted zones of 2D graphene nanolayers, a simple yet effective synthetic protocol for the formation of such intricate nanostructures has been studied by hydrothermal cutting of agro-industrial wastes (AW) and categorized into three major parts: crystal reorientation, hydrothermal cutting, and nanoconstriction formation. As reported in an earlier study,^52^ agro-industrial wastes are composed of a combination of major amorphous carbon phases with small crystalline domains. Hydrothermally treating AW resulted in a drastic decrease of cyclohexane monoclinic carbon phases, giving rise to largely graphene hexagonal phases and textured growth. The crystal reorientation from amorphous carbon phases towards graphene hexagonal phases is attributable to the dominating periodic arrangement of atoms under autogenous pressure and controlled temperature conditions.^52^ Here, Raman spectroscopy was employed to confirm the presence of graphene, depicting typical graphene features in most regions: the G band around 1580 cm^−1^, signifying the E_2g_ phonon at the Brillouin zone (BZ) centre, and the D band around 1360 cm^−1^ due to the breathing modes of 6-atom rings (Fig. 1a).^67,68^ A broad hump at ∼2670 cm^−1^ characteristic of the 2D band is also evident in Fig. 1a. Intriguingly, the second-order Raman scattering process involving two intervalley inelastic scatterings with iTO phonon near the *K* point can be effectively interpreted to estimate the number of layers in graphene by scrutinizing the line shape and intensity.^69^ The broadened 2D band indicates the presence of multi-layer graphene.^70^ As a result, there are numerous possible double resonance scattering processes for 2D bands in multi-layer graphene. The 2D band located at ∼2670 cm^−1^ can be fitted by four Lorentzian peaks for AB stacked bilayer graphene^71^ (Fig. 1b (2D deconvoluted)). The wide band of the 2D peak in the Raman spectra can be attributed to the increase in defects and disorders caused by the formation of in-plane nanogaps on graphene layers^72^ (owing to hydrothermal-cutting of epoxy groups) during the autogenous pressurized hydrothermal-carbonization process.^73^ Interestingly, a band at ∼3300 cm^−1^ appears denoting the second order of D′ band termed as the 2D′ peak (Fig. 1c). Although this particular peak was less studied in the past, it originates from a Raman scattering process where momentum conservation is obtained by the participation of two phonons with opposite wave vectors to further confirm the presence of bilayer graphene.^74^ On further region-specific inspection of the sample using Raman spectroscopy, we found contrasting results, wherein both G and D bands possessed sizeable linewidths. This may be attributed to a conspicuous disorder in the latter case justified by the tendency of both G and D band linewidths to increase monotonously with disorder within the graphene matrix, although not in the same way since the G band could be affected by merging with the D′ band (Fig. 1d).^75^ The D′ band originates from a double resonance process; however, the double-resonance process is within the same Dirac cone unlike the D band for which the double resonance occurs in adjacent *K* and *K*′ Dirac cones in the Brillouin zone. Hence, it can be used to probe the nature of defects through the intensity criterion (Fig. 1e (G/D′ deconvoluted)).^68,76^

**Scheme 1:**
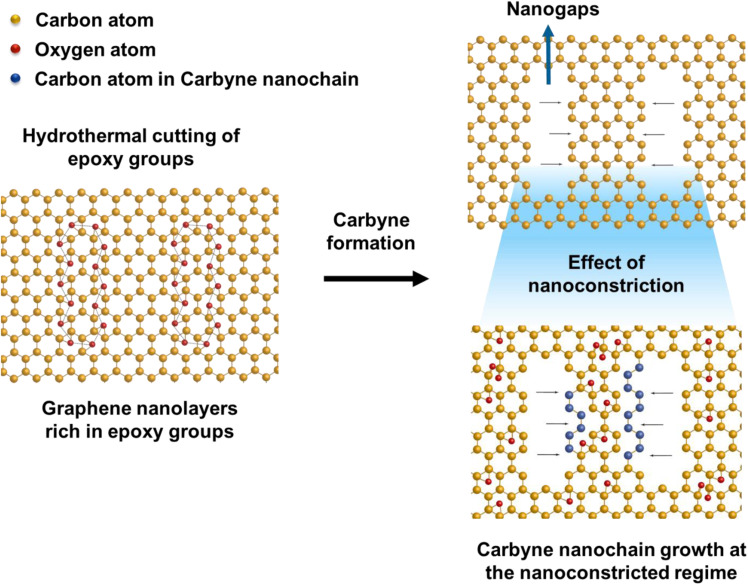
Schematic illustration of carbyne chain formation from the hydrothermal cutting of epoxy-rich graphene layers.

The presence of highly defective graphene in certain regions leads to non-hexagonal sp^2^ defects, which can be attributed to the occurrence of intrinsic defects that were formed to accommodate the edge states and strains.^77^ With narrowing nanoconstriction regions, energetically stable elongated carbyne chains may transpire from 2D graphene nanolayers. This accounts for the emergence of a stark carbyne peak while also retaining G and D bands indicative of disordered graphene. Bolstering the same, we observe a drop in *I*(D) in the Raman spectra (Fig. 1d), corresponding to an increase in the number of defects in graphene alongside a sharp decrease in *I*(2D), which can also be used as an indicator of disordered graphene.^78^ Remarkably, the emergence of a sharp Raman peak at ∼2100 cm^−1^ is observed around the disordered graphene regions generated by carbon triple bonds and offers strong evidence for the presence of carbyne crystals.^29^ The full-widths at half maximum (FWHM) value of this Raman peak becomes narrow, corresponding to the high purity and crystallinity of the carbyne nanocrystals that can be asserted to its significant presence in the sample. Notably, the characteristic carbyne peak (Fig. 1f) existing around 2145 cm^−1^ (FWHM = 7.966) affirms the presence of carbyne nanocrystals (peak width stretching around ∼30 cm^−1^ vibrations). These results were in good agreement with the photoluminescence spectra of the obtained Cp (Fig. S10†). The formation mechanism of carbyne nanochains from epoxy-rich graphene nanolayers were shown in Scheme 1.

### 3.2 Morphological characteristics of the nanomaterial

**Fig. 2:**
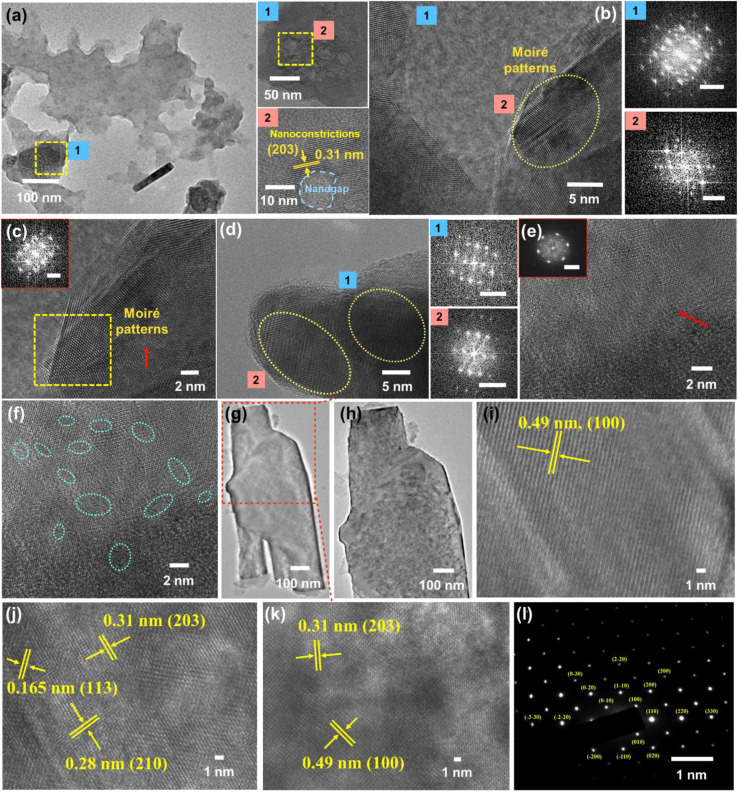
(a) HRTEM images representing the orientation of layered holey graphene sheets (insets 1 and 2) depicting highly porous holey graphene regions with nanoconstricted lattice fringes, followed by (b–f) superimposed and close-packed sheets with maximized areal molecular density representing stacked graphene moiré patterns (with clear moiré images highlighted in (c)) and its corresponding FFT images ((b–e) scale bar: 5 nm^−1^). Further, the hierarchical nano-to-micro (g–k) structures are represented from scrolled layers of graphene (g) to the presence of intrinsic carbyne nanocrystals decoded from its characteristic hexagonal lattice packing system with the corresponding SAED pattern (l).

Corresponding to the contrasting compositional regions indicated by Raman spectroscopy, we performed high-resolution transmission electron microscopy (HRTEM) to recognize the basis of carbyne formation from graphene-rich AB bilayer sheets and its underlying mechanism. Consistent with the Raman findings, layered and highly porous holey graphene sheets guiding the origin of nanoconstricted lattices were observed in Fig. 2a (insets showing nanoconstrictions (1 and 2)). On further inspection of the graphene-rich areas, we found superimposed and close-packed sheets with maximized areal molecular density representing stacked moiré patterns (Fig. 2b–d). Few degrees of twist arising between the assembly of stacked layers of graphene nanosheets gives rise to moiré patterns, and such intricate domains show the existence of nanoconstrictions, which act as a preferential site for the formation of 1D carbyne nanochains.^79^ The magnified views of a selected region of Fig. 2a and FFT patterns corresponding to occurrences of unique lattice fringes are shown in Fig. 2b–d. HRTEM images at these edges of graphene demonstrate that there exist parallel fringes (Fig. 2b and c) and hexagonal patterns (Fig. 2d and e), which are possibly interrelated and originate through a similar mechanism: while the hexagonal patterns are from vdW interactions of graphene layers with rotational mismatches that are vertical to the microscope lens, the observation of parallel fringes comes from the projection of a similar superstructure but with a slight tilt in plane. A closer inspection of Fig. 2c also reveals variation in the widths of the stacking boundaries, indicating the probable occurrence of nanometre-wide transition areas between stacking domains. Moreover, we observe that these stacking boundaries typically consist of random alignments and exhibit a larger extent of regional contrast wherein few regions appear lighter than others, representing that the stacked boundaries can have distinct morphologies based on their specific synthesis conditions. Meanwhile, the light lines are absent in regions of graphene stacking. This selective induction of intensity decline at stacking regions can be described by the obstruction of electron beam diffraction during transmittance through bilayer graphene. Since HRTEM images are generated by electron scattering, the regional intensity is largely based on the diffraction peaks being utilized, consequent to a periodic lattice. In the case of bilayer graphene, as opposed to monolayer graphene, two layers assume a similar crystal orientation, leading to diffraction interference of layers with one another. Thus, the HRTEM intensity is influenced by phase differences or lattice periodicity shifts between the electron wave scattering by bilayer graphene.^80,81^ Intriguingly, the edges of these graphene layers depict a clear formation of (highlighted region in Fig. 2c) moiré patterns. The reduced image contrast at the stacking boundaries (dark lines in Fig. 2b–e), thus, indicates a phase difference in the transition areas (Δ*x*_1_ is nonzero). We propose that this shift in phase can be explained by either relative shifting continuously between the layers due to strain on one of the layers or it can exist due to reconstructed graphene domains attributable to the formation of sharper stacking boundaries. Moreover, contrast variability in images insinuates a difference in the amounts of the shift in the lattice at the transitional areas, which may lead to different morphologies.

### 3.3 Interfacial electronic properties of the material

**Fig. 3:**
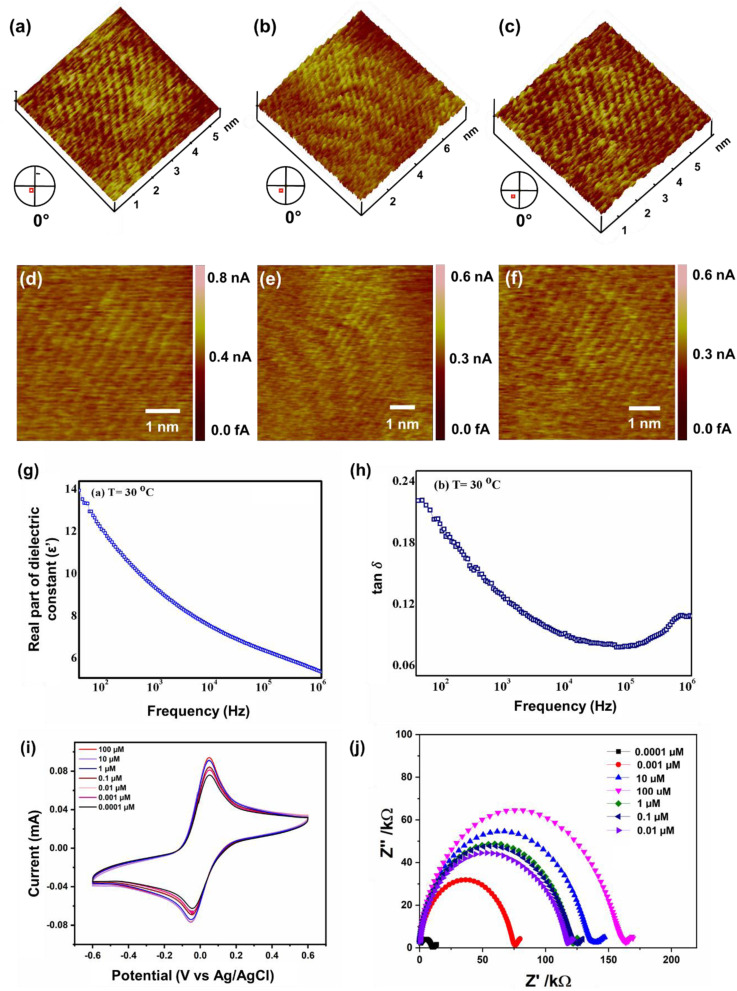
(a–f) Topographical orientations of graphene moiré patterns measured using scanning tunnelling microscopy. (g and h) Dielectric spectroscopic analysis of (g) the localization of electrons, (h) interfacial polarization effect, CV measurements of (i) Cp/SPCE at various concentrations of As^3+^ from 0.0001 μM to 100 μM with 5 mM K_3_Fe(CN)_6_/5 mM K_4_Fe(CN)_6_ as the electrolyte solution at a scan rate of 50 mV s^−1^, (j) EIS analysis of Cp representing the wide range of sensitivity depicting very high sensitivity even at the lowermost As^3+^ concentration.

Several topographical orientations of graphene moiré patterns with preferable defect densities were investigated using Scanning Tunneling Microscopy (STM) (Fig. 3a–f). To detect the magnetic fingerprints and electronic distribution states on graphene moiré patterns, differential conductance was observed at ambient conditions using STM, restricting their distribution closer to the Fermi energy (bias voltage of 20 mV). The STM measurements featuring d*I*/d*V* spectra probe the local density of electronic states (LDOS) along with the generation of periodic potential for graphene that can also be analysed for the determination of surface states of Dirac fermions. A sequence of STM measurements was conducted to analyse the interatomic distances at different parts of the observed moiré patterns since there is a variation in lattice extension for selective domain areas with respect to the other parts of the moiré pattern. A higher order of symmetry in the distribution of d*I*/d*V* curves can be attributed to the nearest-neighbour interlayer coupling induced by the hopping of electrons at the graphene sublattices breaking electron–hole symmetry. Generally, at the higher-energy regimes, the spectroscopy (d*I*/d*V*) tends to be smoothened, minimizing the graphene quasiparticle excitations, which eventually affects the identification of dips over d*I*/d*V* curves. Henceforth, we focused only on the low-energy regimes of graphene moiré patterns where the intervalley scattering gets neglected, showing the uniform distribution of dips along with apparent current density ranging from 0.01–0.03 nA, as shown in Fig. S1a–c.† The d*I*/d*V* curves were linear for most of the region (small d*I*/d*V* gradient), indicating a relatively small variation in LDOS that can be ascribed to the metallic nature in the closer proximity of defect centers. From the STM measurements, the origin of topological defects can be examined *via* typical meadows/cavities within the highly oriented protrusions (3D images) in a wide bias range (−400 mV to +400 mV), which strongly depends on the generated tunnelling current (*i.e.*, from the tip–sample distance). The STM image in Fig. 3a–f shows a transition of crystalline ‘ripple’ domain boundaries to an extended regime of topological defects that can be attributed to the delocalized conduction electrons in graphene moiré edges and the presence of several magnetic impurities localized near the defect centers. The left corner of Fig. 3a, possessing a large protrusion in the form of a hillock, corresponds to the origin of defective sites leading to intricate vacancies majorly influenced by the lattice strain caused by out-of-plane carbon adatoms. Atomic force microscopy (AFM) also demonstrates the ‘ripple’ domains of graphene moiré edges (Fig. S1d†). The migration of carbon adatoms that are oriented in an out-of-plane symmetry from the graphene basal plane can affect the original planar structure to form a bridge configuration (on the top of the C–C bond in the basal plane).^82^ Subsequently, the change in the hybridization state at these lattice-constrained regimes can facilitate the intrusive formation of carbon linear chains due to nanoconstriction. The disappearance of periodicity at the edges of crystalline domains observed in Fig. 3a–f can be ascribed to the emergence of the nanoconstriction zone from the defective sites and at the edge states of the moiré pattern. Both the crest and trough regions of STM images (Fig. 3a–f) reveal the sublattice polarization by the arrangement of the uniform honeycomb structure of graphene. Some of the linear atomic orientation of carbyne nanochains with an interatomic distance of 0.31 nm was also observed (Fig. S8†).

### 3.4 Electrochemical sensitivity and performance

Furthermore, the dielectric spectroscopic analysis suggests the accumulation of conduction electrons at the edge of the grain boundaries, improving the overall polarization as well as dielectric permittivity of the nanomaterial. Such predominant localization of electrons (Fig. 3g) and interfacial polarization effect (Fig. 3h) can offer greater potential for the fabrication of tailorable dielectric sensor devices. Later, we extended the scope of the sensor application by engaging the Cp for electrochemical-based As^3+^ sensing technique. By comparative current response, we observed that the bare SPCE current range was only ∼0.081 mA; however, using Cp-modified SPCE resulted in a much higher current profile of 0.091 mA. Subsequently, upon further addition of 100 μM As^3+^ solution, the current response carried a progressive jump to 0.096 mA (Fig. 3i). This phenomenon indicates that the carbyne-modified electrode demonstrated a significant increase in the peak current compared to the bare electrode. The proposed sensor showed a linear range from 0.0001 μM to 100 μM with an increased sensitivity of 0.22 mA μM^−1^. The Nyquist plot of the EIS spectra explains the electron transfer nature of the deposited film. The resistance created in the electrochemical system varies with an increase in the concentration of the analyte (arsenic solution) depending on the mobility of the electrons from the electrode to the electrolyte solution or *vice versa*. The results in Fig. 3j demonstrate that the Cp exhibits lower electron transfer resistance at lower concentrations (0.0001 μM) of the arsenic solution, which enhances the overall electron-transfer kinetics process correlating with higher sensitivity response.

**Fig. 4:**
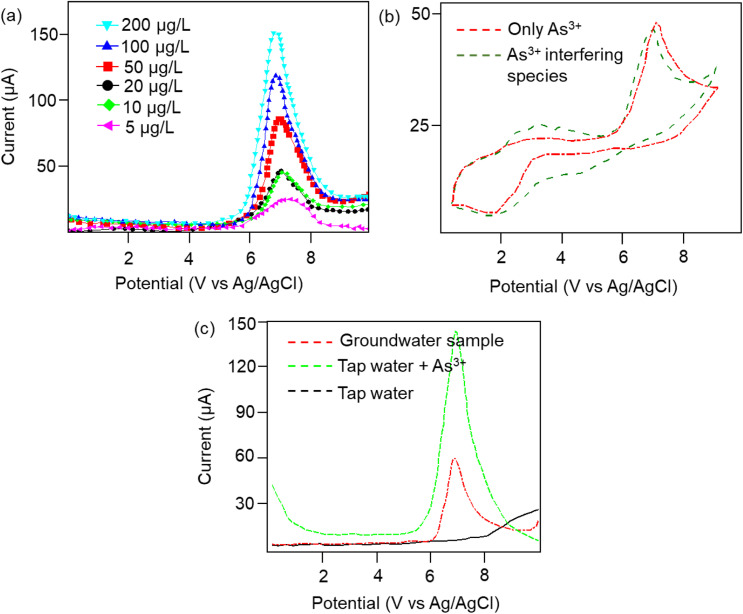
(a) LSV plots of Pt–Cp at different concentrations of As^3+^ (ranging from 5–200 μg L^−1^). (b) CV plots of Pt–Cp for As^3+^ detection in the presence of interfering species (Na^+^, Hg^2+^, Cd^2+^, and Pb^2+^). (c) Real-time sensing of Hindon river water for As^3+^ detection.

Furthermore, to investigate the real-time electrochemical sensing performance, Cp was modified with a Pt electrode (Pt–Cp) and examined by recording the linear sweep voltammetry (LSV) at diverse As^3+^ concentrations (Fig. 4a), detecting the As^3+^ presence in highly interfering electrolyte species (Na^+^, Hg^2+^, Cd^2+^ and Pb^2+^) using CV (Fig. 4b) and real-time sensing of As^3+^ in river water samples (Fig. 4c). LSV plots in Fig. 4a depicted the electrochemical sensing behaviour of various As^3+^ concentrations using the Pt–Cp electrode. Later, CV plots were examined under the same buffer solution composed of other interfering species (Na^+^, Hg^2+^, Cd^2+^ and Pb^2+^) along with As^3+^ species. Intriguingly, there was no pronounced decline in the As^3+^ peak intensity, indicating high selectivity and sensitivity of the electrocatalysts even in the presence of high concentrations of interfering species. A slight change in the peak intensity could be associated with resistance engendered by the larger ionic radii of divalent cations (Pb^2+^, Cd^2+^, Hg^2+^) in the buffer metal ions possessing larger ionic radii generating strong attraction towards the functionalities of Pt–Cp (Fig. 4b). To evaluate its environmental susceptibility in real time, we performed real-time measurements by collecting the groundwater samples at multiple source points along the banks of the Yamuna river (*i.e.* Hindon river traversing through Saharanpur, Muzaffarnagar, Meerut, and Baghpat districts in Uttar Pradesh India). The Hindon river sample was compared with the regular tap water sample. The river sample displayed a concentration of *ca.* 45 μg L^−1^ of As^3+^, while the regular tap water tested from the lab displayed no peak response for As^3+^. Subsequently, 100 μg L^−1^ of As^3+^ was added to the tap water and sensed for the presence of As^3+^*via* electrochemical studies (Fig. 4c). There was a very marginal amperometric drop with a relative standard deviation (RSD) of 2.7% while maintaining 97.3% of the initial peak current response. This indicates the high stability and reproducibility of the modified sensor for real-time As^3+^ determination.

### 3.5 Defect oriented magnetic response and polarization

The low frequency EPR (X-band (9.763 GHz)) technique has been utilized to investigate the origin of highly defective centres of graphene moiré patterns, which not only provides an insight into spin properties but also includes the presence of conduction electrons, unpaired spins, and dangling bonds generated due to the electronic states of different forms of carbon (Fig. S2†).^83,84^ Generally, the intensity of the D band in Raman data can be correlated with the presence of defect centers in the sample. In our experiment, the coupled D and G bands in the Raman spectra lie in accordance with the single broad EPR resonance shown in Fig. S2,† where the contribution of defects to the conductivity is consistent with unique crystallographic and electronic properties as shown in STM results (Fig. S1a–c†). To understand the intricate mechanism of the dependency of carbon-related defects on the microwave power, we determined the saturated behaviour of EPR spectra by qualitatively comparing the relaxation rates of paramagnetic centers keeping the frequency fixed at the resonance frequency of the resonator and sweeping the magnetic field over 200 G. However, only the EPR measurements taken at 2.965 mW indicate the in-phase steady saturation with an inhomogeneous broadening of the Gaussian line shape. Henceforth, the existence of such a single EPR signal can be attributed to the distinct zones of defects formed in the graphene moiré layers, particularly on the surface of graphene planes, edges, in-plane lattice defects along with oxygen-containing functional groups and no significant areas of planar sp^2^ carbon atoms.^1,85^

**Fig. 5:**
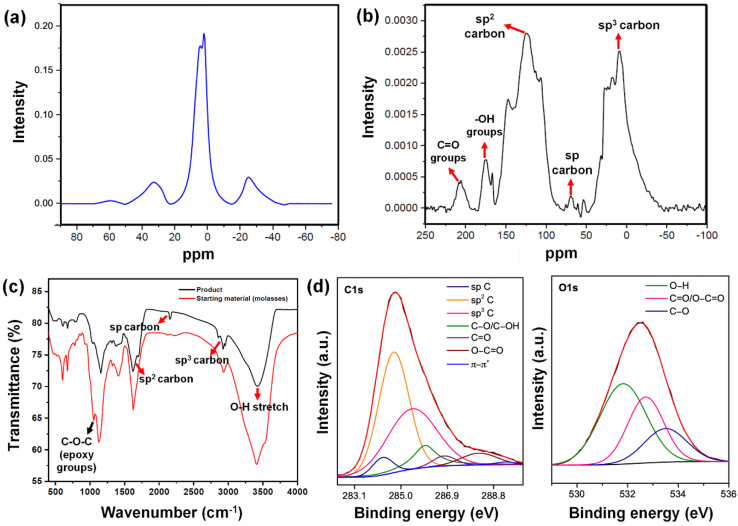
(a) ^1^H NMR spectrum of the Cp. (b) ^13^C NMR spectrum of the Cp. (c) FTIR spectra of the Cp and the starting material (molasses–AW). (d) Deconvoluted C 1s & O 1s XPS spectra of Cp.

Intriguingly, the sp^3^ hybridized defect centers are chemically active, causing strong charge carrier localization that may lead to the enhanced magnetism generated in graphene moiré patterns.^86^ Furthermore, the EPR spectrum and Raman bands follow the Kohn anomaly, which is evident from the broadening of D and G bands, indicating anharmonicity prevailing over the defect-related electron-phonon or phonon-mediated defect (inelastic scattering) interactions.^87^ This phenomenon can be correlated to the increase in the *I*_D_/*I*_G_ ratio from 0.7976 to 0.9532 for the carbyne sample and also lies in accordance with the inelastic scattering of electrons corresponding to iTO phonons (A_1g_ and E_2g_ phonons) near the Brillouin center. The competing spin dynamic mechanisms can be understood *via* analysing the hyperfine splitting with anisotropic *g*-tensors possessing the value of 2.0068, which is quite higher than other related reports,^88–90^ indicating a sequence of a high degree of magnetic interactions from oxygen-centered radicals in association with carbon radicals.^91^ Additionally, the line widths in the EPR signal also confirm the major contribution of paramagnetic centers from the sp^3^ hybridized carbon network as affirmed by the measured value of resonance ranging less than 1 mT of the Gaussian line shape. This is also confirmed by the dramatic shifts in ^1^H NMR peaks indicating paramagnetism (Fig. 5a).^92^

### 3.6 Spectroscopic analysis and MD simulation studies

In addition to the strain-induced lattice shifts and reassembled moiré patterns, we observed several nanogaps in the graphene sheets, giving rise to holey graphene. The ^13^C NMR analysis (Fig. 5b) shows a peak in the region of 124.2 ppm arising due to intact graphene regions. The chemical shift region between 75 and 65 ppm indicates the presence of acetylenic carbon –C≡C–. Furthermore, the NMR signal at around 69 ppm validates the presence of sp carbon, depicting the generation of the carbyne phase. The presence of a peak near 147.2 ppm indicates single vacancy defects in graphene, which reduces the shielding of these carbon nuclei.^93^ Consistent with the holey graphene, sp^3^ carbon peaks centering 9 ppm to 30 ppm are attributable to edge carbon atom chains in graphene generated by hydrothermal cutting. We also observed the retention of ketone groups and hydroxyl groups, which were indicated by the peaks at 205 ppm and 175 ppm, respectively. Bearing in mind the existence of reconstructed graphene domains, these nanogaps uphold the phenomenon of phase difference and strain between layers. The occurrence of the holey graphene layer calls for further mechanistic insights into its formation. The carbon–carbon triple bonds of carbyne (–C≡C– bonds) are commonly identified using Fourier transform infrared (FTIR) spectroscopy. The signal in the FTIR spectrum at ∼2150 cm^−1^ (Fig. 5c) was in good agreement with the previous reports, wherein a similar value for carbon–carbon triple bonds was observed (between 2000 and 2200 cm^−1^).^94^ This phenomenon is a clear identification of the asymmetric C≡C stretching IR vibration bands. Also, these results clearly depicted the presence of carbyne with one-dimensional sp hybridization, consisting of alternating single bonds and triple bonds. The spectra also indicated the presence of other bonds (*e.g.*, epoxy at 1053 cm^−1^, alkoxy C–O at 1153 cm^−1^, and C=O stretch at 1662 cm^−1^, *etc.*). The intensity of the OH band at ∼3414 cm^−1^ is reduced after the hydrothermal treatment, yielding a broad peak at ∼3428 cm^−1^. The FTIR peaks near ∼2925 cm^−1^ and ∼2956 cm^−1^ depicts sp^3^ C–H, corresponding to asymmetrical stretching and symmetrical stretching of methylene groups appearing post hydrothermal treatment. Plausibly, their formation is a consequence of hydrothermal cutting and validates the nanoconstriction phenomenon, finally forming a nanogap. The FTIR results were consistent with results from NMR and Raman spectroscopy. FTIR analyses (Fig. 5c) revealed the disappearance of the vibrational band of epoxy groups at 1054 cm^−1^ in the Cp obtained after the hydrothermal reaction. This can be attributed to the hydrothermal-assisted cleavage of epoxy edges in the precursor, which are fragile and susceptible to attack.^95^ The disappearance of the epoxy band suggests that they break up during the hydrothermal process, through which the bridging O atoms in the epoxy edges are removed. The resultant “unzipped” epoxy edges likely underwent expansion, leading to nanoconstricted areas by the merger of holes facilitated by hydrothermal cutting. The intensity of the OH band at 3414 cm^−1^ is reduced after the hydrothermal treatment, yielding a broad peak at 3428 cm^−1^. The peaks near 2925 cm^−1^ and 2956 cm^−1^, corresponding to asymmetrical stretching and symmetrical stretching of methylene groups appear post hydrothermal treatment. Plausibly, their formation is a consequence of hydrothermal cutting and validates the nanoconstriction phenomenon, finally forming a nanogap.

**Fig. 6:**
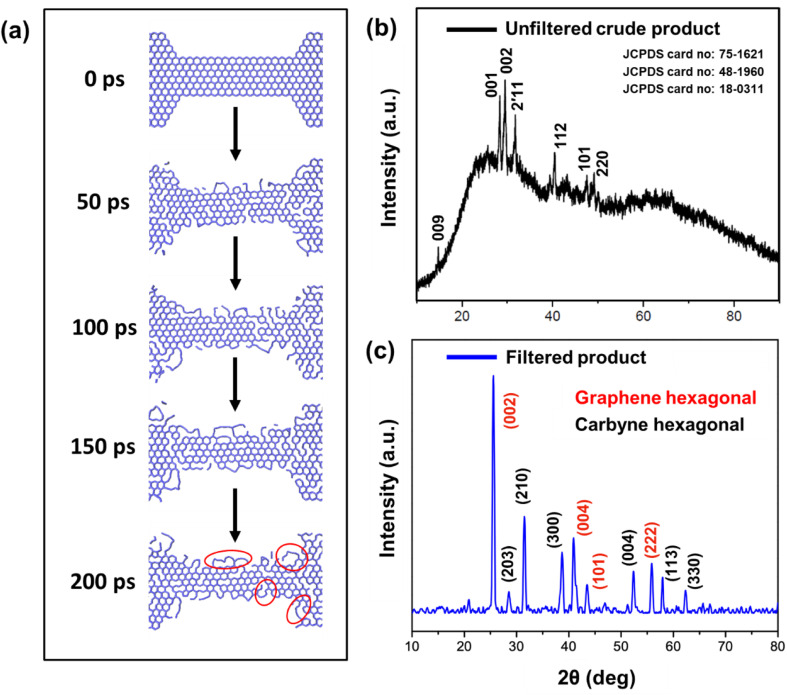
(a) Time course evolution study of the formation of carbyne nanochains from holey graphene strips calculated through MD simulation. The portions circled in red color show bent carbon chains (kinked) formed from graphene. (b) X-ray diffraction analysis for the unfiltered hydrothermal Cp, and (c) XRD patterns of the Cp (carbyne within graphene nanoconstriction) depicting the presence of graphene hexagonal (marked in red colour) and carbyne hexagonal phases (marked in black colour) after filtration treatment.

HRTEM analysis revealed a highly crystalline cylindrical structure along with sheets of graphene in Fig. 2g–i. On closely inspecting the lattice structure, we observed a rectangular arrangement with a lattice spacing of 0.49 nm and (100) plane in one region, indicating crystalline carbyne (Fig. 2i).^29^ In other regions, rectangular and hexagonal lattice spacings of 0.165 nm, 0.31 nm, and 0.28 nm corresponding to planes (113), (203), and (210) confirmed the presence of carbyne (Fig. 2j).^96^ To further analyse the orientation of crystal planes and to interpret the concomitant emergence of carbyne crystals from periodically arranged graphene moiré patterns, XRD analysis was carried out. As shown in Fig. 6b, graphene exhibits a characteristic peak at 2*θ* = 25.58°, depicting the reflection planes (002) of the hexagonal phase of graphene (FWHM = 0.3967), which is correlated to an interlayer spacing of 3.47 Å. These dominating 2D hexagonal reflections were observed using the scattered peaks at 41.43°, 43.55°, and 55.86°, which indexed to (004), (101), and (222) planes, respectively (Fig. 6c). The higher angle (2*θ*) scattered peak at 55.86° with an in-plane lattice spacing of 0.164 nm confirms the stability of the graphene hexagonal phase over the other plausible phases. The narrow width of graphene peaks may be ascribed to two factors: (1) the large sheet size and (2) a relatively high domain order. However, the structural stability of the sp^2^ hybridized system is affected in due course of nanoconstriction experienced at the graphene hexagonal edges. Therefore, the emergence of the sp^2^–sp system is facilitated by nanoconstriction at the basal planes of graphene.

X-ray photoelectron spectroscopy (XPS) and XRD analyses validate the formation of nanogaps in graphene owing to nanoconstriction. In the deconvoluted C 1s spectra (Fig. 5d), fitted with six peaks, the broadening effect of sp^3^ hybridized carbon at 285.4 eV indicates the local rehybridization of carbon atoms from sp^2^ to sp^3^ C atoms. This manifests highly reactive graphene defect sites such as edges, single vacancies, and grain boundaries.^97^ The broadening phenomenon of sp^3^ C atoms (FWHM = 2.39 eV) can be directly correlated to the increase in the concentration of defects,^98^ plausibly causing a zone of nano-constriction, and ultimately nanogaps (Fig. 2a).

The nanoconstriction-facilitated sp^2^–sp emergence, giving rise to carbyne nanocrystals is also indicated by XPS and XRD analyses. The low binding energy peak at 284.2 eV corresponds to the enhanced stability of sp hybridized carbyne nanochains nucleating from the dominating sp^2^ graphitic domains (284.7 eV), where the bridging of sp–sp^2^ clusters is the origin of molecular conductivity.^99^ The XRD pattern shows clean, shaped, strong peaks at 28.54°, 31.49°, 38.71°, 52.87°, 57.91°, and 62.33° depicted by the planes (203), (210), (300), (004), (113), and (330), respectively, which can be indexed to the hexagonal structure of carbyne (Fig. 6c and S7†). The predominance of highly crystalline carbyne peaks over graphene can be attributed to the existence of strong π electron delocalization of sp carbon atoms in comparison to the sp^2^ carbon atoms. Notably, the supremacy of sp^2^–sp hybridization is governed by the intricate Peierls distortion, resulting in the formation of finite carbyne nanochains and decreasing the degree of amorphization of crystalline carbyne domains.^100^ A minimum response of shake-up (0.9% of the total carbon content) of the valence electrons by the core hole expands the possible degeneracy of π sub-bands possessing minimum interband transitions within the confines of the Brillouin zone^101^ and the absence of inter-chain interactions, which is interpreted *via* higher in-plane lattice spacing of 0.148 nm (330) of α-carbyne as observed in XRD. The stability of carbyne nanochains relies concomitantly on the generation of periodic defects in graphene moiré patterns, causing periodic deformation/alteration on the bond lengths of sp-bonded C in the crystal lattices forming internal kinked structures similar to the effect of Peierls transition.^101^ The separation of carbyne chains from nanoconstricted graphene layers was modelled *via* simulation studies and represented using Visual Molecular Dynamics (Fig. 6a, S3 and S4) (ESI Video 1).† Similar to the methodology utilized in previous reports,^60,102^ we have also conducted a 200 ps NVT simulation using the Berendsen–Anderson temperature thermostat with a timestep of 0.2 fs. We also plotted the potential energy of the system with time and percentage of bonds broken to understand the trends in the reaction path (Fig. S3†).

## 4. Conclusion

In conclusion, we equipped a simple yet effective hydrothermal synthesis route using agro-industrial waste molasses to synthesize 1D carbyne nanocrystals within 2D graphene moiré nanolayers as a unique sp–sp^2^ hybridized system. The mechanism of carbyne nanocrystal formation in graphene moiré nanolayers accompanied by holey graphene packets occurs within a nanoconstricted zone, wherein we explored the hydrothermal cutting of epoxy-rich functional groups at graphene nanolayers. By deciphering the topographical properties of the nanomaterial using AFM and STM characterizations along with the dielectric measurements, the rich presence of hybridized defect sites, polarizability, and dielectric permittivity of holey graphene nanolayers with strong charge carrier localization were observed. Selected domains of electron diffraction in HRTEM analysis depicted a rectangular arrangement with a lattice spacing of 0.49 nm and (100) plane, indicating crystalline carbyne presence within graphene nanolayers. Furthermore, XRD patterns confirmed the presence of carbyne nanocrystals with characteristic peak (330) of the α-carbyne phase and dominating reflection planes (002) of the hexagonal phase of graphene (FWHM = 0.3967) with an interlayer spacing of 3.47 Å, ensuring the coexistence of carbyne nanocrystals and graphene moiré nanolayers. The separation of carbyne chains from nanoconstricted graphene layers was modelled *via* MD simulation studies to showcase a clear understanding of carbyne formation from graphene nanoconstriction. Excellent intrinsic properties of Cp, such as predominant localization of electrons, paramagnetic nature, dielectric permittivity, and real-time electrochemical sensitivity, especially in the case of As^3+^ sensing, explore a new arena in the development of sustainable next-generation electronic/sensor devices.

## Data availability

The raw/processed data required to reproduce these findings cannot be shared at this time as the data also forms part of an ongoing study.

## Author contributions

Sampathkumar Jeevanandham: methodology, investigation, visualization, data curation, validation, writing-original draft, writing-review & editing. Dakshi Kochhar: methodology, investigation, writing-original draft. Omnarayan Agrawal: investigation, data curation. Siddhartha Pahari: resources, visualization. Tamal Goswami: software, formal analysis. Chirantan Kar: methodology. Indra Sulania: investigation, validation. Monalisa Mukherjee: conceptualization, supervision, visualization, methodology, writing-original draft, writing-review & editing, project administration, funding acquisition.

## Conflicts of interest

All authors declare no conflicts of interest.

## Supplementary Material

NA-006-D4NA00076E-s001

NA-006-D4NA00076E-s002

NA-006-D4NA00076E-s003
