# Supplementary material for: Unravelling the formation of carbyne nanocrystals from graphene nanoconstrictions through the hydrothermal treatment of agro-industrial waste molasses
Source: Nanoscale Adv. 2024 Mar 28;6(9):2390–406. doi: 10.1039/d4na00076e (PMC11059479; doi:10.1039/d4na00076e)
Supplement: NA-006-D4NA00076E-s001 [file NA-006-D4NA00076E-s001.pdf]

Electronic Supplementary Material (ESI) for Nanoscale Advances.
This journal is © The Royal Society of Chemistry 2024

Supplementary Information

Unravelling the formation of Carbyne Nanocrystals from Graphene nanoconstrictions using hydrothermal treatment of agro-industrial waste molasses

Sampathkumar Jeevanandham,[a] Dakshi Kochhar,[a†] Omnarayan Agrawal,[a†] Siddhartha Pahari,[b] Chirantan Kar,[c] Tamal Goswami,[d] Indra Sulania,[e], Monalisa Mukherjee[*a]*

a. Amity Institute of Click Chemistry Research and Studies, Amity University Uttar Pradesh, Noida 201301, India.

b. Department of Chemical Engineering & Applied Chemistry,200 College Street,Toronto ON M5S 3E5 Canada.

c. Amity Institute of Applied Science, Amity University Kolkata, Kolkata, West Bengal 700135, India.

d. Department of Chemistry, Raiganj University, Raiganj, Uttar Dinajpur, West Bengal 733134, India.

e. Inter University Accelerator Centre, Vasant Kunj, New Delhi, Delhi 110067, India.

**Corresponding author. E-mail: mmukherjee@amity.edu*

†Equal contribution from authors

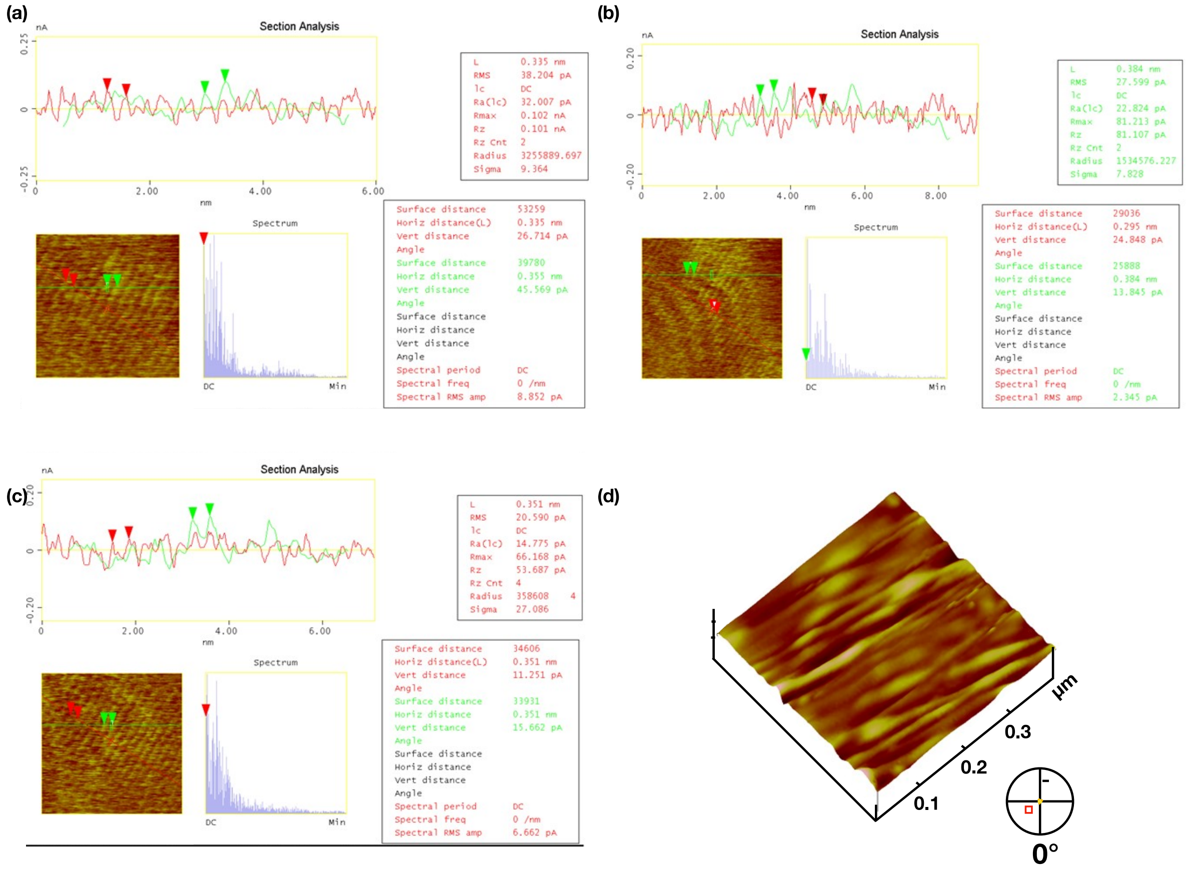

Fig. S1 a-c Distribution of dI/dV curves to analyse the interatomic distances at different parts of the observed Moiré patterns. **d** AFM images of ‘ripple’ domains of graphene moiré edges.

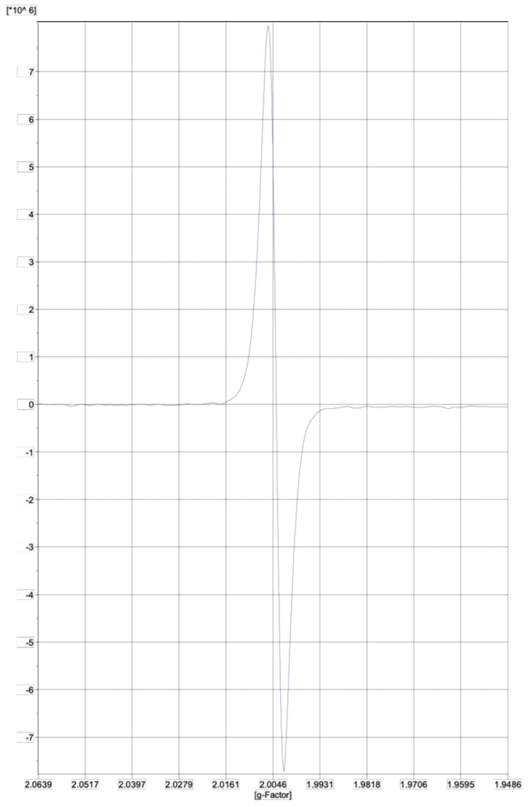

Fig. S2 Low frequency EPR showing paramagnetic centers.

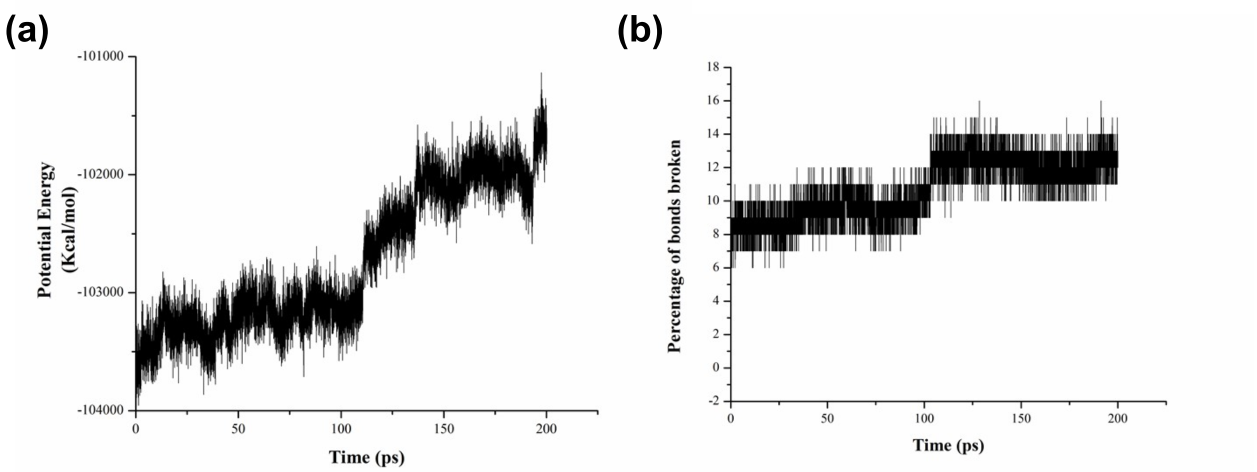

Fig. S3 The variation of potential energy **a** and breaking of bonds **b** during the MD simulation.

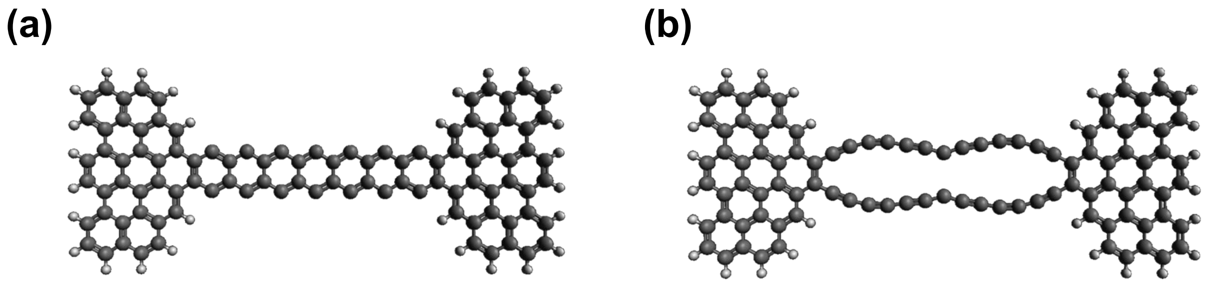

Fig. S4 a Initial geometry considered for modelling the constricted graphene segment and **b** carbyne chain formation from constricted graphene using DFT calculation.

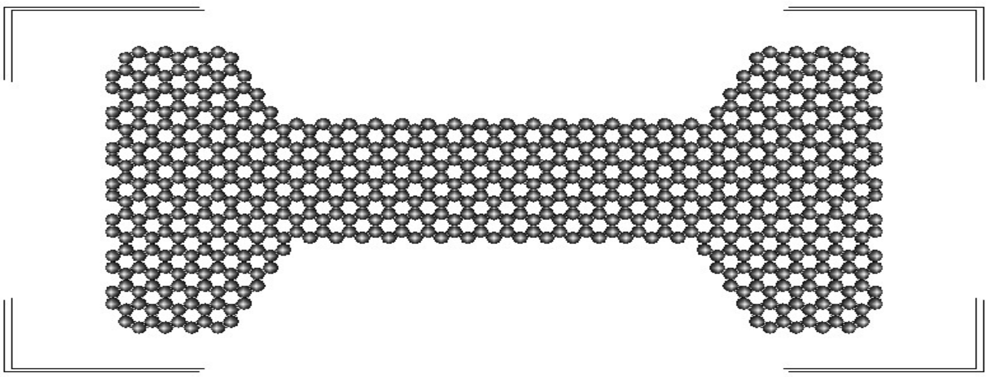

Fig. S5 A single-layer rectangular graphene with carve from the longer end confined in a 75Å × 35Å × 40Å periodic box has been used to construct our simulation system.

Sample Name	C atom %	H atom %	O atom %	N atom %
Starting material	80.21	2.28	16.31	1.20
Product	85.39	6.91	5.41	2.29

Table S1. Elemental analysis of Carbon, Hydrogen, Nitrogen and Oxygen at.% from starting molasses and the product.

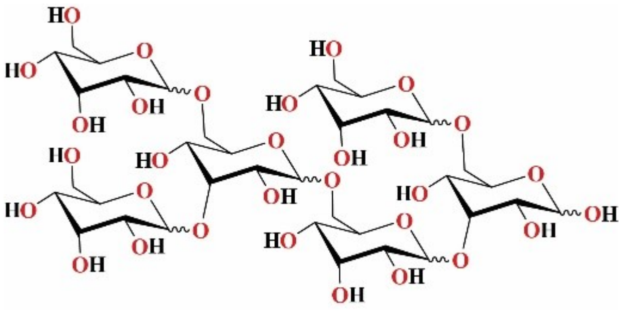

Fig. S6. Structural backbone of starting material (sugarcane waste – molasses)

The structural units of the starting material (molasses) are made of high percentage of invert sugar comprising several functional groups such as -OH, -CH_2OH, etc., (forming intermolecular hydrogen bonding) connected with glycosidic linkage via covalent bonding joining two carbohydrate (sugar) molecules together. Such structural backbone and their respective functional groups which are rich in carbon source are subjected to hydrothermal treatment to generate nanocarbon products.

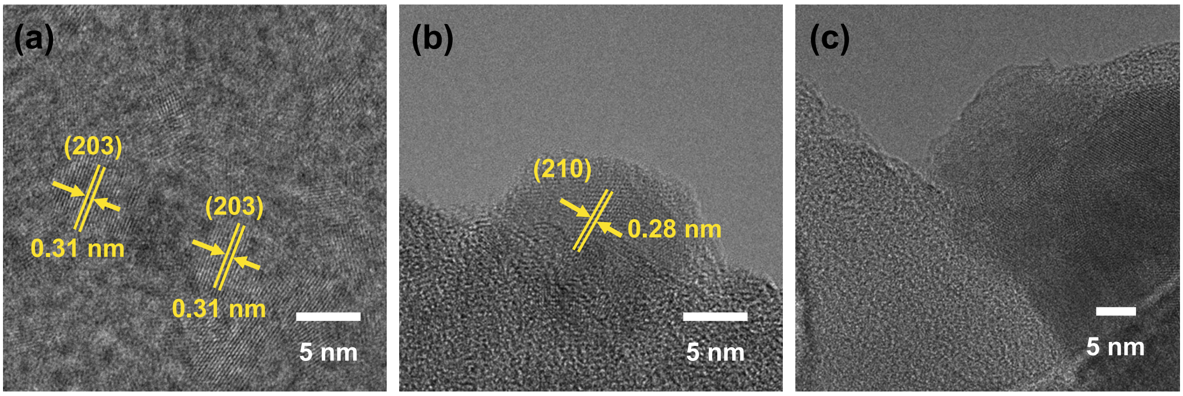

Fig. S7. High magnification HRTEM images of holey graphene sheets at the edges representing the nanoconstricted growth of carbyne nanocrystals (a) on the surface of the sheets, (b) at the edges and (c) at the interfaces.

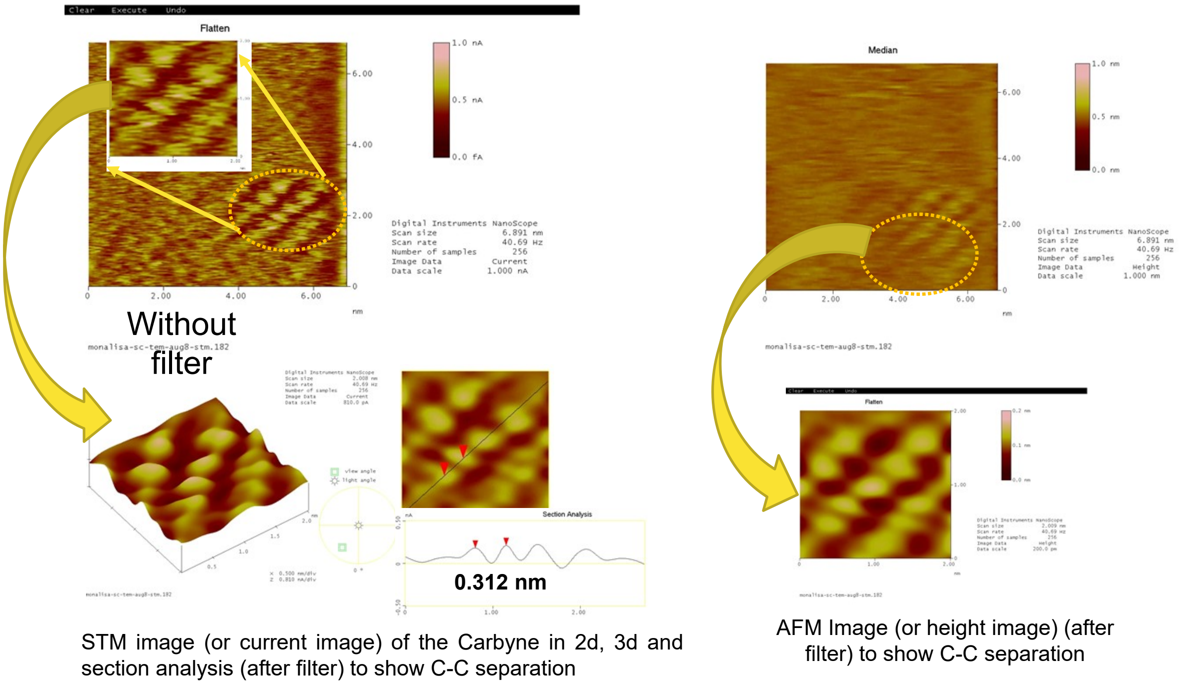

Fig. S8. STM and AFM images of holey graphene sheets without filtered images to differentiate noise and distinct tunnelling current signals (in nA), the interatomic distance of

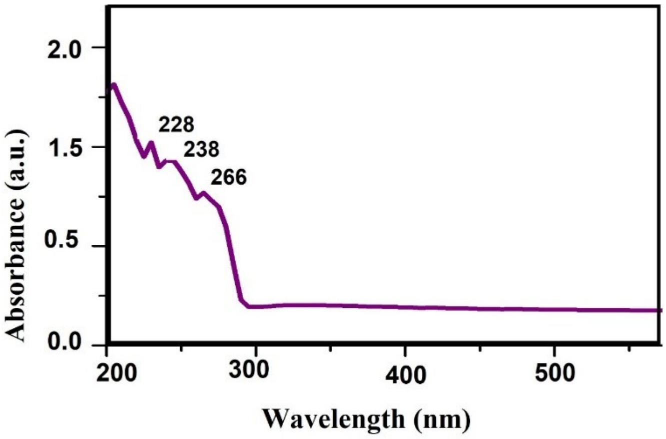

0.31 nm confirms the presence of carbyne.

Fig. S9. UV-vis spectroscopy of the carbyne product.

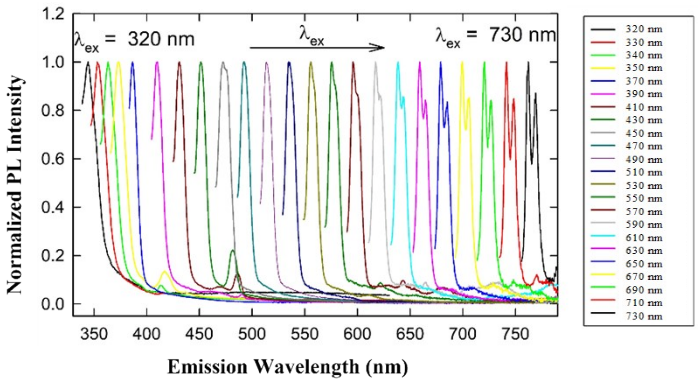

Fig. S10. Photoluminescence spectroscopy of the carbyne product.

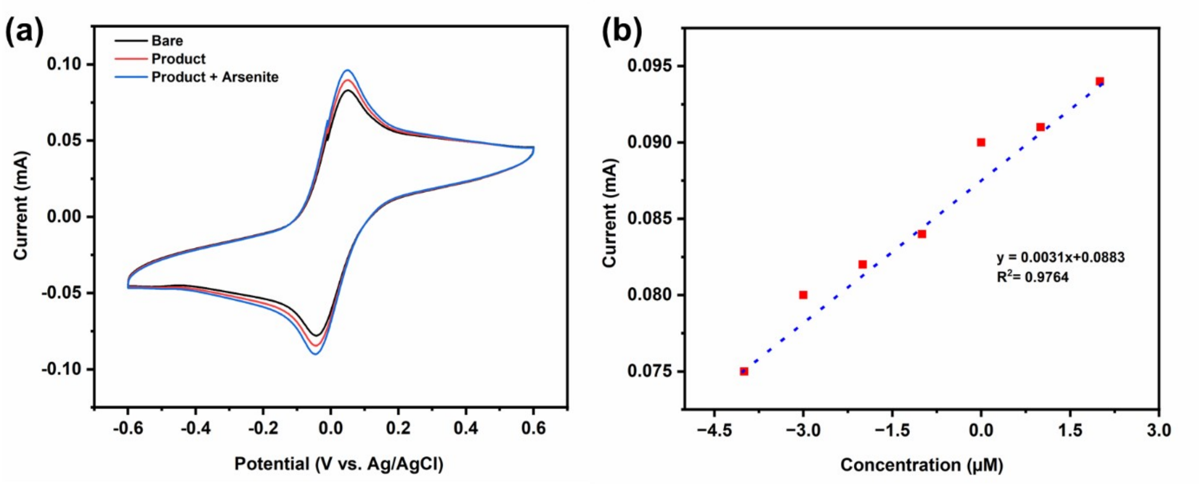

Fig. S11. CV studies were conducted to evaluate the electrochemical response of a product/SPCE towards As (III). (a) CV response obtained after modifying the SPCE with the product in 5mM potassium ferricyanide ($K_3Fe(CN)_6$)/5 mM potassium ferrocyanide ($K_4Fe(CN)_6$) solution with 100 µM concentration of As (III) in the solution (scan rate: 50 mV/s), and (b) product/SPCE show the corresponding variation of current density as a function of the concentration of As(III) solution.

A calibration curve was also generated for arsenic concentrations from 0.0001 µM to 100 µM (**Fig. S11(b)**). The results proved that product/SPCE responds linearly between the working electrode current value and the As(III) concentration. The lower limit of detection (LOD) was determined by the CV was 0.0001 µM. The fitted linear equation obtained was y = 0.0031x+0.0883 with a correlation coefficient of 0.9972.

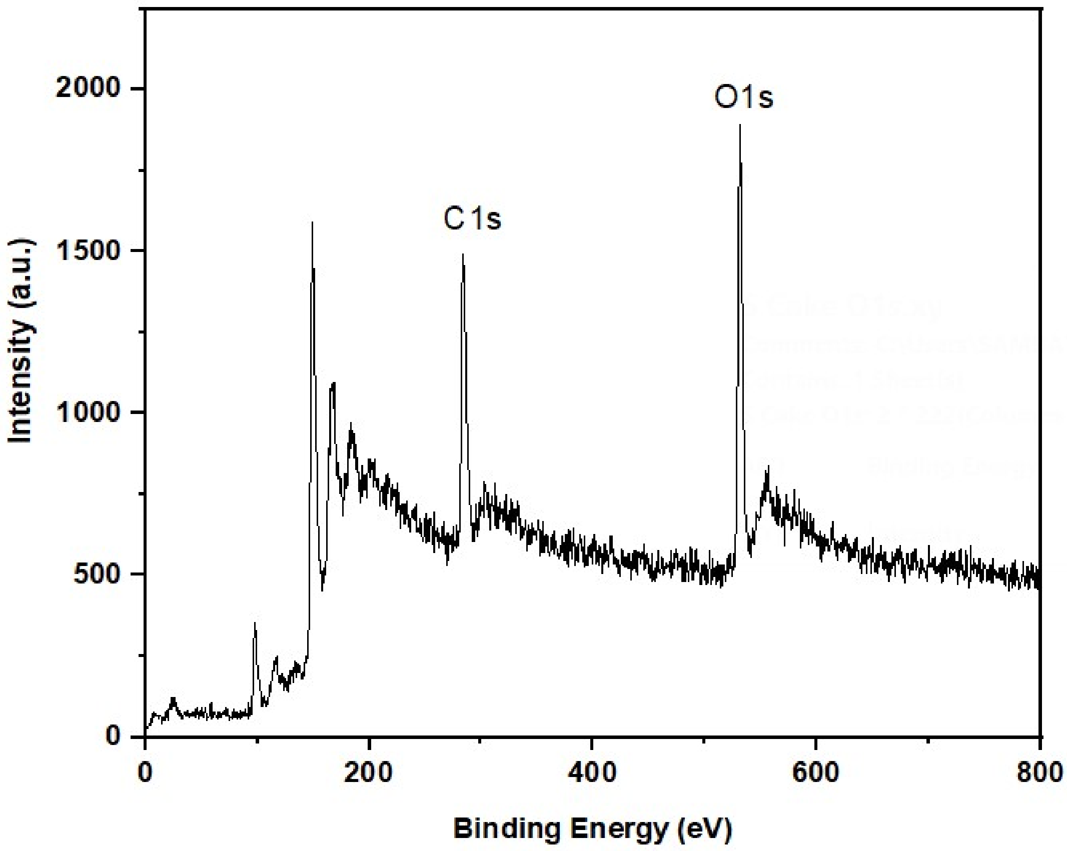

Fig. S12. XPS Survey spectra of the as-synthesized product depicting the absence of N1s spectra.

C1s spectra components	Binding Energy (eV)
sp C	284.2
sp^2C	284.7
sp^3C	285.5
C-O/C-OH	286
C=O	286.8
O-C=O	288.2
π-π^* interactions	289.3

O1s spectra components	Binding Energy (eV)
O-H	531.8
C=O/O-C=O	532.7
C-O	533.5

Table S2. XPS peak fitting components of C1s and O1s spectra.
